# Research Advances in Age-Related Macular Degeneration

**DOI:** 10.3390/jcm11133627

**Published:** 2022-06-23

**Authors:** Solmaz Abdolrahimzadeh

**Affiliations:** 1 Ophthalmology Unit, Neurosciences, Mental Health and Sense Organs (NESMOS) Department, Faculty of Medicine and Psychology, University of Rome Sapienza, 00189 Rome, Italy; solmaz.abdolrahimzadeh@uniroma1.it; 2 Ophthalmology Unit, St. Andrea Hospital, 00189 Rome, Italy

The first descriptions of the condition now known as age-related macular degeneration (AMD) appeared in 1852; however, it is only since the 1970s that our knowledge on AMD has substantially increased [1]. AMD is one of the leading causes of severe visual loss worldwide, with a dramatic effect on the quality of life of patients. Since the introduction of intravitreal vascular endothelial growth factor (VEGF) therapy, there has been a major paradigm shift in the management of exudative AMD in terms of visual function. Furthermore, continuously evolving diagnostic imaging methods in ophthalmology, such as spectral domain optical coherence tomography (SDOCT) and optical coherence tomography angiography (OCTA), enable high-resolution evaluation of macular morphology and greatly facilitate patient follow-up.

Ongoing research aims to better clarify the multifactorial mechanisms involved in the pathogenesis of the disease with emphasis on the search for retinal and choroidal biomarkers that can facilitate clinical assessment, improve early diagnosis, and provide information on the factors involved in disease progression, with the ultimate aim of refining management strategies and improving the quality of life of patients. The present Special Issue in the *Journal of Clinical Medicine* is dedicated to high-quality scientific papers focusing on potential biomarkers, treatment trends, quality of life, and potential clinical challenges in the management of patients with AMD.

Several interesting findings can be derived from this collective body of work. In alignment with the concept of biomarkers, and investigation directed towards understanding disease pathogenesis, Hong et al. used OCTA and structural SDOCT to study retinal vessel density and inner retinal thickness in AMD [2]. Patients with geographic atrophy (GA) in one eye and intermediate AMD in the fellow eye were assessed. The rationale was that, although the outer retina is primarily affected in GA, some studies have shown inner nuclear layer and ganglion cell loss, and reduced density of the superficial vascular plexus (SVP) and deep vascular plexus (DVP) [3,4]. Hong et al. found lower vessel density of the SVP and DVP and thinner ganglion cell, inner plexiform, and outer nuclear layer in eyes with GA. Interestingly, at 2 years of follow-up they found that seven fellow eyes with intermediate AMD developed GA with reduced vessel density of the SVP and DVP, suggesting the role of vessel density and inner retinal layer thickness as potential GA markers in non-exudative AMD.

The role of inner retinal alterations in AMD was also highlighted by Abdolrahimzadeh et al. [5]. An important phenotype in AMD is subretinal drusenoid deposits (SDD) that are located in the subretinal space above the retinal pigment epithelium in contrast to drusen that are localized below the retinal pigment epithelium [6]. GA and type 3 neovascularization together with choroidal thinning are strongly associated with SDD. Abdolrahimzadeh et al. used near-infrared reflectance and SDOCT to evaluate eyes with subretinal drusenoid deposits versus conventional drusen. They found inner retinal layer thinning in the central macula and superior parafoveal area in early/intermediate AMD in eyes with SDD or conventional drusen with respect to age-matched control eyes, further highlighting inner retinal layer thicknesses as potential biomarkers in AMD.

Kim et al. studied the clinical aspects of disease by evaluating the trend of anti-VEGF agent selection for the initial treatment of AMD [7]. As there is no gold standard in the choice of anti-VEGF agents, they found that, in older patients and those with type 3 macular neovascularization, ranibizumab was preferred, whereas in patients with polypoidal choroidal vasculopathy, aflibercept was adopted. The authors concluded that the choice for the use of aflibercept could have been conditioned by previous reports of a higher resolution of polypoid lesions with aflibercept and the thicker choroid in this AMD subgroup [8,9]. In general, since aflibercept induces a higher degree of choroidal thinning than ranibizumab [10], the authors postulate that this could have been the reason behind the choice of ranibizumab in more than half of the patients with type 3 macular neovascularization, where patients are at higher risk for GA and have thinner choroids. A further issue they highlighted was the association of prolonged anti-VEGF therapy and possible cerebrovascular accidents, because aflibercept is associated with a more profound decrease in systemic VEGF levels than ranibizumab, and older age is a risk factor [11].

The recent global health emergency caused by the COVID-19 pandemic induced physicians dealing with intravitreal anti-VEGF therapy to adapt patient management strategies in accordance with government restrictions and emergency hospital organization. Spain was one of the most affected countries in Europe. Valverde-Megías et al. reported on the effect of the COVID-19 lockdown in Spain on structural and functional outcomes of neovascular AMD patients [12]. The authors found that patients with both intra and subretinal fluid on SDOCT were more likely to suffer greater visual loss. Their results were in accordance with the majority of the studies published in 2021 where the COVID-19 emergency had a negative impact on both functional and anatomical parameters in patients with neovascular AMD [13]. The work of Valverde-Megias et al. indicates that treatment should be a priority in patients at high risk in emergency situations.

Another aspect to consider when evaluating patients with AMD is the quality of life. Hara et al. developed a questionnaire survey on driving among patients with AMD in Japan [14]. Central macular impairment and central scotomas reduce visual acuity and contrast sensitivity in AMD. Since driving is an important aspect of daily life, the authors evaluated driving capabilities in patients with exudative AMD causing unilateral blindness or paracentral scotoma without vision deterioration. They found that that 66% of patients were still driving; moreover, >50% of patients continued driving even with the knowledge of the dangers and risks. Their results were consistent with those reported in the MARINA and ANCHOR studies [15]. The authors discussed that there is a higher number of vehicle incidents in adults above 65 years with respect to other age groups [14]. Although multiple factors could be involved, such as age-related physical changes and delayed response to braking and steering, a study showed that AMD patients required additional time to recognize signals and had delayed brake responses with respect to normal controls [16]. Thus, multidisciplinary medical evaluation is warranted to consider the specific risks during driving in patients with AMD.

The data, recommendations, and scientific evidence collected for this Special Issue highlight that, although we have come a long way in terms of diagnosis, classification, and management in patients with AMD, this pathology still remains a challenge for physicians. Given the contributions regarding the various aspects of research in AMD, it is clear that further investigation on AMD is necessary and will continue to flourish. As the Guest Editor, I would like to acknowledge and thank the authors for their valuable contributions, the reviewers for their precious work, and the members of the *JCM* Editorial Office for their constant support.
